# Tree species occurring in Amazonian wetland forests consistently show broader range sizes and niche breadths than trees in upland forests

**DOI:** 10.1002/ece3.11230

**Published:** 2024-04-25

**Authors:** Bruno Garcia Luize, Clarisse Palma‐Silva, Tadeu Siqueira, Thiago Sanna Freire Silva

**Affiliations:** ^1^ Departamento de Ecologia, Instituto de Biociências Universidade Estadual Paulista (UNESP) Rio Claro Brazil; ^2^ Departamento de Biologia Vegetal, Instituto de Biologia Universidade Estadual de Campinas – UNICAMP Campinas Brazil; ^3^ School of Biological Sciences University of Canterbury Christchurch New Zealand; ^4^ Biological and Environmental Sciences, Faculty of Natural Sciences University of Stirling Stirling UK

**Keywords:** extent of occurrence, flood, hydrological niche, lowlands, macroecology, neotropics, niche tolerance, species distribution, vascular plants

## Abstract

Generally, species with broad niches also show large range sizes. We investigated the relationship between hydrological niche breadth and geographic range size for Amazonian tree species seeking to understand the role of habitat specialization to Amazonian wetlands and upland forests on the current distribution of tree species. We obtained 571,092 valid occurrence points from GBIF and SpeciesLink to estimate the range size and the niche breadth of 76% of all known Amazonian tree species (5150 tree species). Hydrological niche breadth was measured on different unidimensional axes defined by (1) total annual precipitation; (2) precipitation seasonality; (3) actual evapotranspiration; and (4) water table depth. Geographic range sizes were estimated using alpha‐hull adjustments. General linear models were used to relate niche breadth to range size while contrasting tree species occurring and not occurring in wetlands. The hydrological niche breadth of Amazonian tree species varied mostly along the water table depth axis. The average range size for an Amazonian tree species was 751,000 km^2^ (median of 154,000 km^2^ and standard deviation of 1,550,000 km^2^). Niche breadth–range size relationships for Amazonian tree species were positive for all models, and the explanatory power of the models improved when including whether a species occurred in wetlands or in terrestrial uplands. Wetland species had steeper positive slopes for the niche breadth–range size relationship, and consistently larger range sizes for a given niche breadth. Amazonian tree species varied strongly in hydrological niche breadth and range size, but most species had narrow niche breadths and range sizes. Our results suggest that the South American riverscape may have been acting as a corridor for species dispersal in the Neotropical lowlands.

## Graphical abstract

The general ecological pattern for a positive range size‐niche breadth association holds for Amazonian tree species. Furthermore, those wetland‐adapted tree species have broader niche breadth and large range sizes compared with tree species only occurring in upland forests.

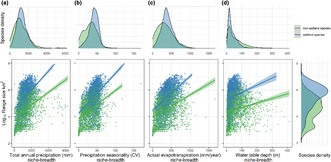

## 1 INTRODUCTION

Species niche breadths and geographic ranges tend to evolve together, producing an ecological pattern where species with larger niche breadths are likely to also have larger geographic distributions (Sexton et al., 2017; Slatyer et al., 2013). The lack of effective geographic barriers for plant dispersal (Dexter et al., 2017; Nazareno et al., 2017) and the moderate topographic and climatic gradients along most of the Amazonian region may have promoted copious dispersal of species through the landscape, leading to large range sizes. Indeed, several Amazonian tree species expanded their distributions beyond the tropical rainforest biome, making the Amazon a source of species for other neotropical biomes (Antonelli et al., 2018). The wide ranges of plant lineages with distributions centered on the Amazonian lowlands (Dexter et al., 2017) may have resulted from species evolving broader niches as they interacted with the climatic and physiographic features of the Amazonian lowlands. But species also tend to conserve their niches, and wide ranges do not necessarily link with broader niches.

Andes orogeny has long been recognized for contributing both directly and indirectly to Neotropical plant species diversity (Gentry, 1982; Hoorn, Wesselingh, ter Steege, et al., 2010). These contributions include changes in lowland hydrography (Hoorn & Wesselingh, 2010) and climate (mainly precipitation patterns, Insel et al., 2010), and forcing species migrations toward the Amazon lowlands (Gentry, 1982; Householder et al., 2016). Tropical mountains have thus been suggested as evolutionary cradles (Hoorn et al., 2013), promoting rapid speciation through specialization along the short‐range, conspicuous environmental gradients that follow elevation (Gentry, 1982; Janzen, 1967). Mountain uplifting also had a direct influence on species distributions by creating effective geographic barriers, and species with distributions skewed toward the Andes are likely to have relatively narrow niche breadths and geographic ranges (Hoorn et al., 2013). Remarkably, apart from Andean orogeny few other historical factors have been raised to date to explain the origin of the enormous diversity of plants across the Amazonian lowlands. In contrast to montane habitats, the lowlands have been extensively present across space and time and may harbor 33% of all Neotropical plant species diversity (Gentry, 1982). Recent studies suggest almost 14,000 seed plant species, of which 6727 are trees (Cardoso et al., 2017).

One remarkable feature of the lowlands that has been present throughout geological time in the Amazon lowlands is the large extent of wetland habitats, covering areas much larger than montane habitats (above 500 m altitude), with likely historical implications for the biota. The vast South American river network and associated wetland extent have influenced continental physiography at least since the Lower Miocene (Hoorn & Wesselingh, 2010). Wetlands covered an area larger than 1.5 × 10^6^ km^2^ of the former Amazon basin throughout the Miocene, comprising much of the present‐day western Amazonian lowlands and being possibly the largest and longest‐lived wetland system in Earth's geological history (Hoorn, Wesselingh, Hovikoski, & Guerrero, 2010; Latrubesse et al., 2010). To this day, South America still has some of the largest extents of wetlands worldwide (Gumbricht et al., 2017), and over half of the known Amazonian tree species (3615 spp.) are known to occur in wetland habitats (Luize et al., 2018). Considering the prevalence and extent of wetlands in the Amazonian lowlands through time and the large proportion of the world's richest tree flora adapted to waterlogged habitats, it is therefore reasonable to expect a considerable influence of Amazonian wetlands on tree species diversification and dispersal (i.e., niche breadth and range size) through deep time.

The hyper‐seasonality observed in Amazonian wetlands (e.g., long‐standing floods of c.a. 6 months and upper than 5 m of water column) is regarded as a harsh environment, demanding very specific ecophysiological adaptations for tree survival (Parolin et al., 2004) and likely promoting habitat specialization (Wittmann et al., 2013). However, high climatic seasonality (e.g., floods and droughts), low energy availability (e.g., water uptake for photosynthesis), or environmental extremes (e.g., heat waves in relatively open habitats) can also select for higher tolerances to environmental extremes and lead to wider niches and larger range sizes, as shown for tree species in North America (Morin & Lechowicz, 2013). We can thus hypothesize that once Amazonian species developed the necessary traits to colonize wetlands, they were able to increase their geographic ranges, covering much of the wet climates found in the Americas. Additionally, wetland trees in the Amazon experience annual, long‐lasting inundations that prevent tree root respiration and reduce water uptake. This seasonal flooding stress may act functionally in a similar way to seasonal drought stress (Parolin et al., 2010), perhaps enabling wetland species to also colonize dryer Neotropical biomes such as Cerrado and Seasonally Dry Forests.

In this study, we investigate the relationship between niche breadth and geographic range size for 5150 Amazonian tree species. As waterlogged habitats are regarded as more extreme habitats for tree species to colonize, we postulate two opposing hypotheses: (H1) tree species able to occur in wetlands have broader niches and larger geographic ranges than tree species occurring only in terrestrial (upland) environments—that is, wetland tree species tend to be generalists; (H2) tree species able to occur in wetlands have narrower niches and smaller range sizes compared with those not occurring in wetlands—that is, wetland tree species tend to be specialists. To test these hypotheses, we derived continental range size and hydrological niche breadth estimates for Amazonian tree species and estimated the relationship between these variables, accounting for the ability or not of each species to colonize wetland habitats.

## 2 METHODS

### 2.1 Data acquisition

**FIGURE 1:**
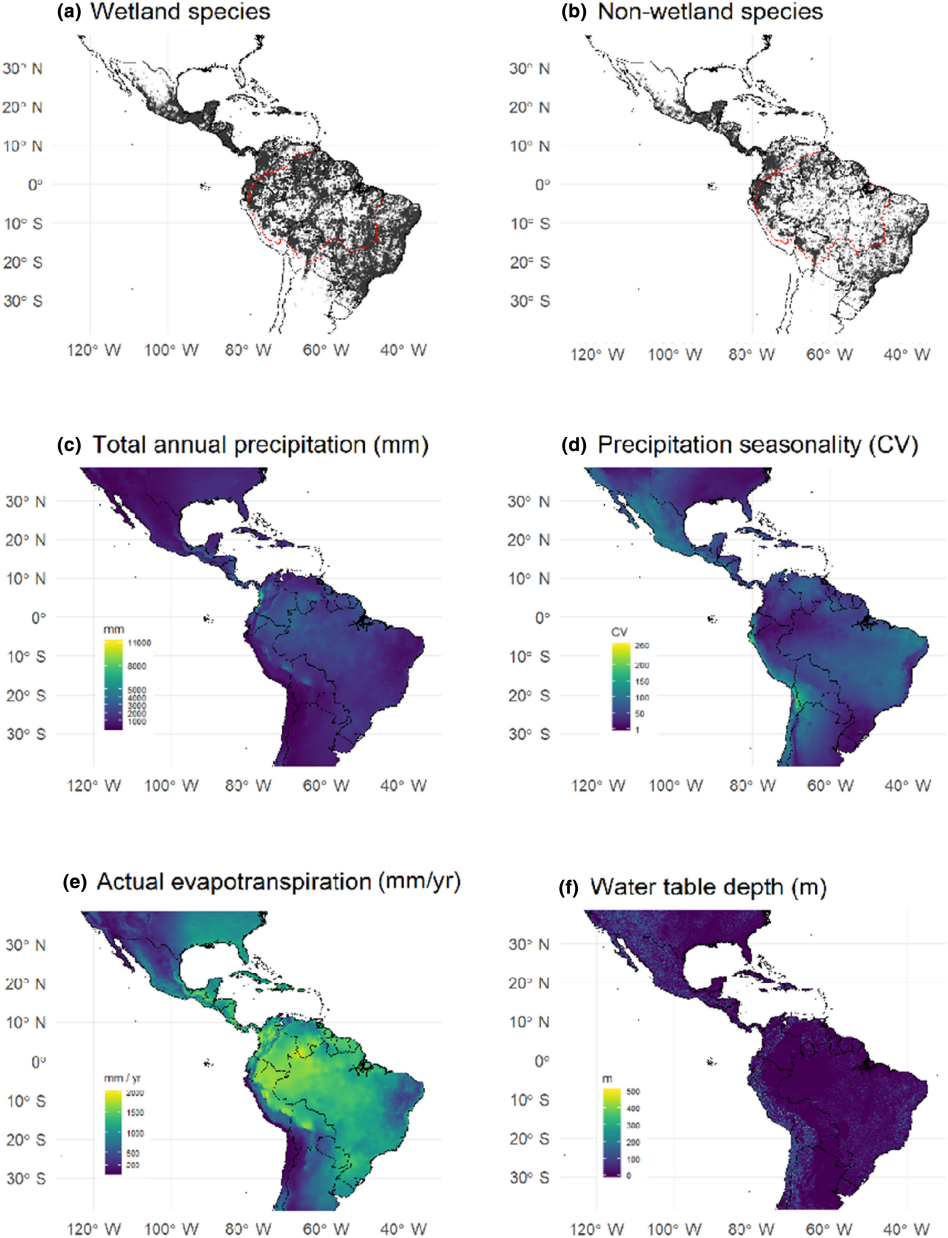
Map of the Americas bound by latitudes 35° N and S: (a) geolocated records of Amazonian tree species that do occur in wetlands (black dots); (b) geolocated records of Amazonian tree species that do not occur in wetlands. In (a) and (b) the red line shows the boundaries of Amazonia sensu stricto. (c) Total annual precipitation (mm), (d) precipitation seasonality (CV), (e) actual evapotranspiration (mm/year), and (f) water table depth (m below land surface).

We queried the GBIF (GBIF.org, 2018) and SpeciesLink (specieslink.net, 2018) databases for occurrence records of all preserved vascular plant specimens recorded between 1970 and 2017 across the Americas. The search returned 6,528,962 records from GBIF and 3,877,675 from SpeciesLink. We merged the two sets of records and filtered the results by matching species names to the taxonomic vetted list of 6727 tree species that have been confirmed for the Amazonian lowlands (Cardoso et al., 2017). The remaining records included only specimens already logged with their most up‐to‐date scientific names in GBIF and SpeciesLink, not including occurrence records for specimens labeled as synonyms or misspelled to ensure taxonomic correctness. We then used the workflow implemented in the “speciesgeocodeR” package (Töpel et al., 2016) of the R language (R Core Team, 2018) to remove occurrence records with geographic issues. Specifically, we removed all records (i) outside terrestrial limits, (ii) with missing values in the coordinates, (iii) non‐valid coordinates, (iv) coordinates that are equal zero, (v) latitude equal longitude, and (vi) records located up to 0.5 degrees from country capitals. We also excluded species with less than three occurrence records given limitations to estimate range sizes using alpha hull. Our final dataset comprised 571,092 occurrence records in the Americas and included 5150 tree species. Finally, we classified each of the 5150 tree species into those occurring in Amazonian wetlands and those not occurring in Amazonian wetlands (i.e., only recorded in upland forests). This classification was done based on the tree species list presented in Luize et al. (2018), which is a review of species lists from tree inventories and botanical collections performed in Amazonian wetland habitats. Tree species listed as occurring in Amazonian wetland yielded a total of 461,666 occurrence records and 2838 species (Figure 1a); species that does not occur in wetlands yielded a total of 109,426 occurrence records and 2312 species (Figure 1b). Note that species occurring in wetlands can also have occurrence records in upland areas (i.e., we are not able to identify wetland‐exclusive species due to the lack of consistent habitat metadata on species records, see Luize et al., 2018).

To describe the hydric conditions found across the Americas, we chose four environmental variables, comprising three climatic and one edaphic variable. The first two climatic variables were determined using historical averages of total annual precipitation (mm) (Figure 1c) and precipitation seasonality (coefficient of variation) (Figure 1d) from the WorldClim v.1.4 database (i.e., bio12 and bio15, Hijmans et al., 2005). Total annual precipitation estimates the expected amount of water reaching the soil per year, and precipitation seasonality indicates the likelihood of seasonal water deficit. The third climatic condition was the average actual evapotranspiration (mm/year, Figure 1e), between 1950 and 2000, from the CGIAR‐CSI soil‐water balance model (Trabucco & Zomer, 2010). Actual evapotranspiration characterizes a climatic condition of water‐energy balance that is closer to real water availability to plants than precipitation alone (Stephenson, 1990). Together, these three climatic conditions approximate the amount and variability of plant water supply in time and space. As an edaphic environmental condition, we used the results from a global model for water table depth (m below land surface) (Figure 1f) that is constrained by ground observations and calibrated by climate, terrain, and sea level (Fan et al., 2013). All environmental information was obtained as grid layers with cells in the most detailed spatial resolution available (i.e., equal to 30 arc‐seconds or c.a. 1 km^2^ at Equatorial latitudes).

### 2.2 Species niche breadths

To measure niche breadth, we extracted the raw and standardized *z*‐values of the cells intersecting species occurrences for each selected environmental grid layer, using the function “extract” from the R package “raster” (Hijmans & van Etten, 2014). To compute the standardized *z*‐values, we cropped each grid layer for the extended tropical American region (bound by 35° N and S), and then applied a standard *z‐*normalization (*z = x*_*i*_ − *μ/σ*, where: *x*_*i*_ is the grid cell value, and *μ* and *σ* are the sample mean and standard deviation of all grid cells across the region). We repeated this procedure for each selected environmental layer. Then, we computed species niche breadths as the univariate interquartile range between the 10th and 90th quantiles for each of the four environmental conditions. We chose interquartile distance rather than minimum and maximum values to reduce the influence of extreme environmental conditions that could arise from remaining geolocation errors or wrongful environmental estimates. Thereby, our estimate of niche breadth relies on the distance between two points within the range of environmental values a species can occur, regardless of the number of records available for each species.

### 2.3 Species geographic range sizes

Geographic range sizes were calculated by fitting an α‐convex hull to the occurrence records of each species across the entire tropical range of the Neotropics, as this method has been successfully used to measure and compare species ranges (Gallagher, 2016). The α‐convex hull algorithm is based on the Voronoi diagram and Delaunay triangulation of spatial coordinates points and is suitable only when there are more than three georeferenced occurrence points (Pateiro‐López & Rodríguez‐Casal, 2010). To estimate the α‐convex hull for each tree species, we used the function “EOO.computing” of the “ConR” R package (Dauby et al., 2017), which also imports functions from the package “alphahull” (Pateiro‐López & Rodríguez‐Casal, 2010). The method implemented in “ConR” produces estimates biased toward wider distributions, but it is the standard method used to assess the conservation status following IUCN red‐list standards (Dauby et al., 2017). We first fit α‐hulls using five different values of the α parameter (α = {0.5, 1, 3, 5, 10}) and the default package value for the α buffer (0.1°) for all tree species. A value of α close to 0 will simply correspond to a distance buffer around each occurrence record (i.e., locality), while α tending to infinity will lead to a generalized convex hull (i.e., generality), encompassing all point coordinates (Gallagher, 2016). For tree species with occurrence records following a straight line, range size was computed by building a buffer polygon of 0.1° width around the line segment (Dauby et al., 2017). After visually inspecting the resulting hulls together with the occurrence records and plotting the overall range size distribution from each parameter combination, we selected α = 3 as the parameter with the best compromise between locality and generality for estimating range size for all species. Areas were computed geodetically in reference to the WGS 84 ellipsoid.

### 2.4 Niche breadth and range size relationships

We evaluated separately the overall relationship between each species niche breadth dimension and range size, and the effect of wetland adaptations, using a series of linear models.

(1) $$\log_{10}\left(\mathrm{RS}\right)=\alpha +\beta_{1}\mathrm{NB}\pm \varepsilon$$

(2) $$\log_{10}\left(\mathrm{RS}\right)=\alpha +\beta_{1}\mathrm{NB}+\beta_{2}\left(W\vee \mathrm{NW}\right)\pm \varepsilon$$

(3) $$\log_{10}\left(\mathrm{RS}\right)=\alpha +\beta_{1}\mathrm{NB}+\beta_{2}\left(\mathrm{NW}\right)+\beta_{3}\mathrm{NB}\left(W\vee \mathrm{NW}\right)\pm \varepsilon$$

where RS is range size; NB is the measured niche breadth for each of the four studied niche dimensions; W is the set of tree species classified as occurring in wetlands and NW is the set of tree species that does not occur in wetlands; and *α*, *β*, and *ε* are the estimated parameters of the models, respectively, the intercept, slope, and remaining deviance. To reduce non‐linearity and heteroscedasticity, we applied a base‐10 logarithmic transformation to species range sizes. As the expected relationship between niche breadth and range size is positive, we compared the effect size of contrasting models using standardized slope coefficients. We used *F*‐tests and the Akaike information criterion (AIC) to compare the competing models.

Previous studies on the relationship between niche breadth and range size suggest that the existence of phylogenetic signals in both species attributes can lead to the overestimation of the relationship (Dexter & Chave, 2016; Sexton et al., 2017). To ensure that the strength of the relationship between niche breadth and geographic range size was not overestimated, we implemented phylogenetic generalized least squares models to control for phylogenetic affinities among species. The phylogenetic generalized least squares models were fitted for Equations (1), (2), (3) using the “pgls” function implemented in the “caper” R package (Orme et al., 2023). The phylogenetic hypothesis applied to consider the phylogenetic non‐independence between species attributes was built using the “V.phylomaker” R package (Jin & Qian, 2019) to prune species present in the GBOTB phylogeny (Smith & Brown, 2018). Species lacking phylogenetic information were bound to the phylogeny using Scenario.3 of V.phylomaker. The slope coefficients and the adjusted *R* ^2^ of the linear models were compared to the respective values of the models controlling for the phylogenetic non‐independence of species attributes.

Since species niche breadths and range sizes were calculated using the same occurrence records, estimates of the niche breadth vs. range size relationship were not completely independent. Each estimate is based on a separate set of records (niche breadth uses all records while range size considers only the outermost records within the occurrence range), a remaining bias may still affect the estimations. To quantify this possible circularity bias, we applied a randomization procedure with 500 iterations to produce independent estimates of the relationship between range size and niche breadth measurements (Rocha et al., 2018; Siqueira et al., 2009). For each iteration, species occurrence records were split randomly into two independent sets of equal size; the first half was used to determine species range size and the second half to measure species niche breadth, using the same procedure described previously. To refrain from obtaining spurious estimates, this randomization was applied only for tree species with more than 10 occurrence records (4239 species). We then computed 500 coefficient estimates for all general linear models (Equations (1), (2), (3)) using the independently derived variables. The difference between model slopes computed for all available occurrence records (*β*_all_) and the model slopes computed from the randomized sets (*β*_rand_) allowed the evaluation of the bias arising from lack of independence.

To determine which niche breadth dimension had the greatest explanatory power when modeling Amazonian tree species range size, we fitted a multiple linear model with all niche variables.

(4) $$\log_{10}\left(\mathrm{RS}\right)=\alpha +\beta_{1}{\mathrm{NB}}_{\mathrm{TAP}}+\beta_{2}{\mathrm{NB}}_{\mathrm{PS}}+\beta_{3}{\mathrm{NB}}_{\mathrm{AET}}+\beta_{4}{\mathrm{NB}}_{\mathrm{WTD}}\pm \varepsilon$$

where TAP is total annual precipitation, PS is precipitation seasonality, AET is actual evapotranspiration, and WTD is water table depth. We then partitioned the total explained variance of the model following the approach by Lindeman et al. (1980) as implemented on the “relaimpo” R package (Grömping, 2006). This approach decomposes *R* ^2^ into non‐negative contributions to the multiple linear model. The order of the explanatory variables in the model is permuted and the average of each variable's contribution is computed over the different sets of models, without weighting the explanatory variables among different models (Grömping, 2006). Before applying variance partitioning, we assessed the correlation between niche breadth measurements and found a moderate correlation for most of the variable comparisons with a maximum Pearson's *r* coefficient of .65 between niche breadth for precipitation seasonality and actual evapotranspiration.

## 3 RESULTS

### 3.1 Distribution of hydrological niche breadths for Amazonian tree species

The niche breadth of Amazonian tree species varied the most along the axis of water table depth (range = 0–410 m; *z*‐values = 0–9.9; coefficient of variation = 93.8%). The niche breadth axis for total annual precipitation ranged between 0 and 6252 mm (*z*‐values = 0–7.2; cv = 54.2%), followed by actual evapotranspiration (range = 0–1670 mm/year; *z*‐values = 0–3.9; cv = 50.8%), and precipitation seasonality (range = 0–170 SD; *z*‐values = 0–7.2; cv = 43.0%). A total of 65 species had the measured niche breadth for at least one dimension equal to 0: 23 for total annual precipitation (20 Not occurring in wetlands NW, 3 occurring in Wetlands W), 53 for precipitation seasonality (50 NW, 3 W), 20 for actual evapotranspiration (18 NW, 2 W), and 31 for water table depth (26 NW, 5 W). In general, species with zero niche breadth had less than five records but there was one species not occurring in wetlands (*Guarea zepivae* T.D. Penn.) with a total of 11 occurrence records that still yielded a niche breadth of zero for precipitation seasonality. Niche breadth was weakly related to the number of available occurrence records used for its estimation (*r* = .23 NB_TAP_; *r* = .32 NB_PS_; *r* = .30 NB_AET_; *r* = .09 NB_WTD_).

### 3.2 Distribution of range sizes for Amazonian tree species

The number of occurrence records available to estimate range size was highly variable, with 51 tree species having the minimum necessary three points of occurrence, whereas 437 tree species had ≥300 records. The maximum number of records (4059) was observed for *Myrcia splendens* (Sw.) DC., native from South and North America. The range size estimated for each species was strongly related to the number of occurrence records used for estimation (*r* = .85), and the distribution of range sizes for Amazonian tree species varied from 370 to 16,560,000 km^2^, with a skew toward small ranges but clearly showing species with very large ranges.

### 3.3 Niche breadth–range size relationships for Amazonian tree species

**FIGURE 2:**
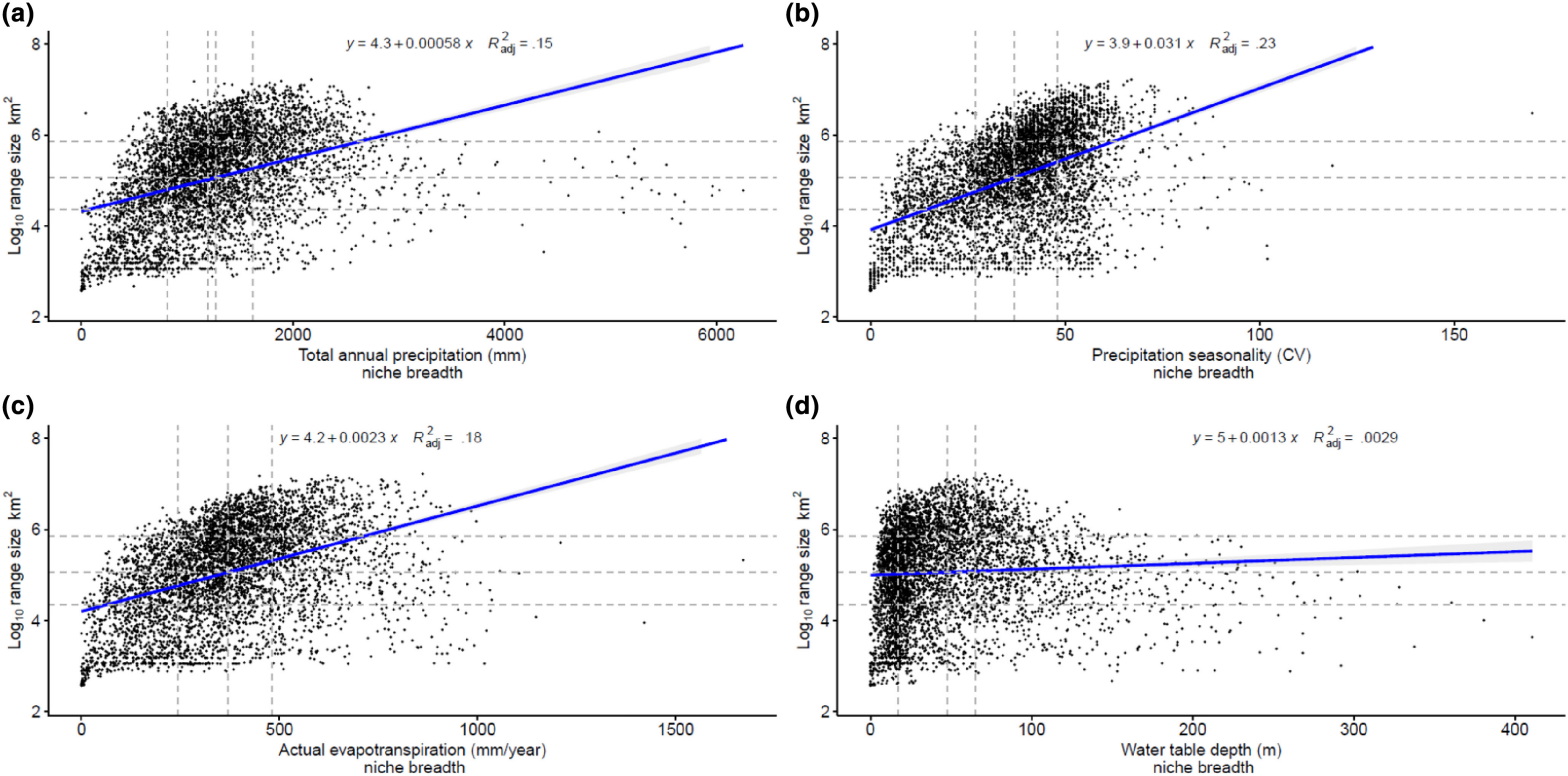
Relationship between niche breadth and range size (logarithmic scale) for 5150 Amazonian tree species on four different niche breadth axes: (a) total annual precipitation, (b) precipitation seasonality, (c) actual evapotranspiration, and (d) water table depth. All models show a positive relation between niche breadth and range size. Dashed horizontal lines show the mean, and the first and third quartiles for the logarithmic distribution of range size and the distribution of niche breadths.

All niche breadth variables were positively related to species range size (Figure 2, Table 1). The steepest model slope was observed when niche breadth was characterized using precipitation seasonality (*β*_std_ = 0.48, $R_{\mathrm{adj}}^{2}$ = .23, Figure 2b), followed by actual evapotranspiration (*β*_std_ = 0.42, $R_{\mathrm{adj}}^{2}$ = .18, Figure 2c), total annual precipitation (*β*_std_ = 0.39, $R_{\mathrm{adj}}^{2}$ = .15, Figure 2a), and water table depth (*β*_std_ = 0.06, $R_{\mathrm{adj}}^{2}$ = .003, Figure 2d). The linear models controlling for the phylogenetic signal in species range size and niche breadth produced higher slope values than those observed for the simple linear models (Table 2). The increased slope values for the phylogenetic controlled relationship between range size and total annual precipitation niche breadth (*β*_std_ = 0.59, $R_{\mathrm{adj}}^{2}$ = .33), precipitation seasonality niche breadth (*β*_std_ = 0.49, $R_{\mathrm{adj}}^{2}$ = .10), actual evapotranspiration niche breadth (*β*_std_ = 0.64, $R_{\mathrm{adj}}^{2}$ = .25) and water table depth niche breadth (*β*_std_ = 0.26, $R_{\mathrm{adj}}^{2}$ = .09) suggest an even stronger positive relationship between range size and niche breadth.

**TABLE 1:** Estimated parameters for the generalized linear models relating to niche breadth and the base‐10 logarithm of range sizes for Amazonian tree species.

Niche breadth	*α* (± SD)	*β*_1_ (± SD)	*β*_2_ (± SD)	*β*_3_ (± SD)	*R* ^2^ ($R_{\mathrm{adj}}^{2}$)	*F*‐statistic	AIC	ΔAIC
Annual precipitation	4.321 (0.027)	0.0006 (1.9e‐05)	—	—	.15 (.15)	919.3	14,096	1416
	3.843 (0.027)	0.0005 (1.6e‐05)	0.92 (2.3e‐02)	—	.34 (.34)	1384	12,727	47.66
	3.957 (0.031)	0.0004 (2.1e‐05)	0.61 (4.9e‐02)	0.00024 (3.4e‐05)	.35 (.35)	948.3	12,679	0
Precipitation seasonality	3.909 (0.031)	0.0312 (7.9e‐04)	—	—	.23 (.23)	1543	13,592	828.14
	3.758 (0.029)	0.0245 (7.6e‐04)	0.72 (2.4e‐02)	—	.34 (.34)	1333	12,795	31.38
	3.875 (0.036)	0.0208 (9.9e‐04)	0.38 (6.3e‐02)	0.00902 (1.5e‐03)	.34 (.34)	905.2	12,763	0
Actual evapotranspiration	4.199 (0.028)	0.0023 (6.9e‐05)	—	—	.18 (.18)	1133	13,918	1195.87
	3.844 (0.027)	0.0020 (6.2e‐05)	0.84 (2.3e‐02)	—	.34 (.34)	1354	12,767	45.04
	3.965 (0.032)	0.0016 (8.0e‐05)	0.52 (5.2e‐02)	0.00086 (1.2e‐04)	.35 (.35)	926.5	12,722	0
Water table depth	4.998 (0.020)	0.0012 (3.2e‐04)	—	—	.0031 (.0029)	16.13	14,926	1394.95
	4.300 (0.025)	0.0039 (2.8e‐04)	1.03 (2.5e‐02)	—	.23 (.23)	808	13,538	6.83
	4.332 (0.027)	0.0034 (3.3e‐04)	0.95 (3.8e‐02)	0.00192 (6.4e‐04)	.24 (.23)	542.4	13,531	0

*Note*: The intercept and slopes for each model (Equations (1), (2), (3)), standard deviance, *F*‐statistic, AIC, and ΔAIC values are for comparison among the models. *p*‐Values were <.001 for all models.

**TABLE 2:** Estimated parameters for the phylogenetic generalized linear models relating to niche breadth and the base‐10 logarithm of range sizes for Amazonian tree species.

Niche breadth (phylogenetic GLMs)	*α* (± SD)	*β*_1_ (± SD)	*β*_2_ (± SD)	*β*_3_ (± SD)	*R* ^2^ ($R_{\mathrm{adj}}^{2}$)	*F*‐statistic	AIC	ΔAIC
Annual precipitation	3.780 (2.909)	0.0008 (1.7417E‐05)	—	—	.33 (.33)	2614	27,093	2436
	3.559 (2.307)	0.00075 (1.40E‐05)	0.8617 (0.015)	—	.58 (.58)	3623	24,685	28
	3.609 (2.29)	0.0006 (2.11E‐05)	0.59 (0.055)	0.00019 (3.5E‐05)	.58 (.58)	2439	24,657	0
Precipitation seasonality	3.883 (3.383)	0.0319 (0.0013)	—	—	.10 (.10)	593.9	28,646	1739
	4.154 (2.857)	0.0108 (0.0011)	0.9368 (0.0205)	—	.36 (.36)	1451	26,907	0
	4.105 (2.858)	0.0124 (0.0023)	1.0143 (0.0971)	−0.0021 (0.0026)	.36 (.36)	967.7	26,908	1
Actual evapotranspiration	3.657 (3.079)	0.0035 (8.2E‐05)	—	—	.25 (.25)	1784	27,677	1731
	3.675 (2.622)	0.00243 (7.4E‐05)	0.8135 (0.0018)	—	.46 (.46)	2203	26,025	79
	3.252 (2.603)	0.0036 (0.00015)	1.3506 (0.062)	−0.0015 (0.00016)	.46 (.46)	1519	25,946	0
Water table depth	4.590 (3.405)	0.0061 (0.0002)	—	—	.09 (.09)	516	28,715	4827
	3.640 (2.146)	0.0116 (0.00018)	1.3425 (0.0151)	—	.63 (.63)	4559	23,960	72
	3.769 (2.131)	0.0100 (0.0002)	1.1004 (0.0319)	0.0037 (0.0004)	.64 (.64)	3107	23,888	0

*Note*: The intercept and slopes for each model, standard deviance, *F*‐statistic, AIC, and ΔAIC values are for comparison among the models. *p*‐Values were <.001 for all models.

The lack of independence between estimations of niche breadth and range size did not change the observed positive relationship between niche breadth and range size (Figures S1–S4), but slopes estimated using randomizations showed that lack of independence may lead to both slope underestimation and overestimation (Figures S1 and S2). The maximum difference between slopes estimated with the randomization procedure and the observed *β*_std_ slopes was small (*β*_std_ difference = −0.31), with most *β*_std_ differences ≤ |0.03|. In general, slopes obtained by randomization were higher than slopes obtained using all available occurrence records (Figure S2). The only exception was the model for NB_TAP_ where slopes estimated using independent sets were lower than observed slopes (Figure S1).

### 3.4 The role of wetland adaptations on the niche breadth–range size relationship of Amazonian species

All general linear models yielded *p*‐values lower than .001 as expected from the large sample sizes (Table 1). The lowest support for an estimated niche breadth–range size relationship was found for water table depth (*p* = .00006, Table 1). The models that included an interaction term between niche breadth and wetland occurrence (Equation 3) had the lowest AIC values (Table 1), despite the larger number of estimated parameters. The simpler generalized linear models (Table 1) produced smaller AICs when compared with the respective AICs for the phylogenetic generalized linear models (Table 2). Nevertheless, among the phylogenetic generalized linear models, those including the interaction term between niche breadth and wetland occurrence were the ones producing the smaller AICs, except for the relationship between range size and niche breadth as measured for precipitation seasonality, which showed the smaller AIC for the model not including the interaction term (Table 2).

**FIGURE 3:**
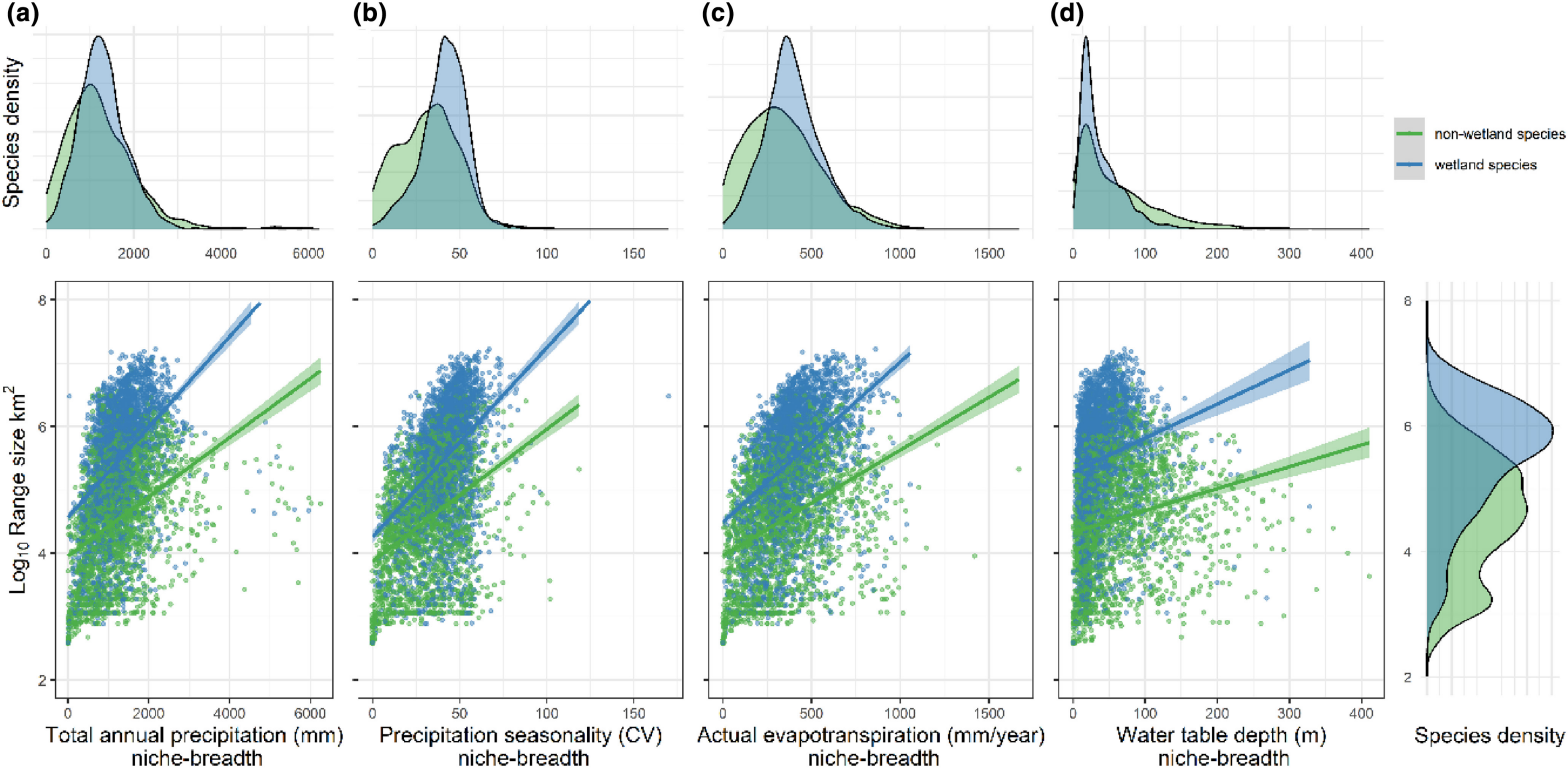
Relationship between niche breadth and range size (logarithmic scale) for Amazonian tree species that occur in Amazonian wetlands (*n* = 2838 species) versus species that do not occur in Amazonian wetlands (*n* = 2312 species). Linear models are shown for four different niche breadth axes: (a) total annual precipitation; (b) precipitation seasonality; (c) annual actual evapotranspiration; and (d) water table depth. The fitted curves for each group follow the model in Equation 3.

The inclusion of wetland/non‐wetland species as an explanatory variable yielded higher intercepts and slightly steeper slopes for tree species occurring in wetland than for species that do not occur in wetlands (Figure 3 and Table 1). The strongest difference in intercept and slope between species groups was observed when using water table depth as an explanatory variable, followed by actual evapotranspiration (Table 1). All models showed a relatively steeper increase in the logarithm of range size along the gradient of niche breadth (i.e., steeper slope) for wetland tree species when compared with non‐wetland species.

Variance partitioning among all four niche variables showed that precipitation seasonality had the highest explanatory power (12%), followed by actual evapotranspiration (7.5%) and total annual precipitation (7.2%), while water table depth had very low explanatory power (0.5%) in estimating the logarithm of species range size.

## 4 DISCUSSION

We provide empirical support for the general ecological pattern of positive relationship between niche breadth and range size (Slatyer et al., 2013), and show that after accounting for phylogenetic affinities among species, the niche breadth–range size relationship of Amazonian trees is even stronger, supporting findings for a coevolution between species range size and niche breadth (Sexton et al., 2017). Furthermore, our results show that tree species occurring in Amazonian wetland forests have a steeper increment in range size for each increment in niche breadth, compared with tree species only occurring in upland forests. The niche breadths and range sizes of Amazonian tree species varied greatly, showing a pattern where most species tolerate moderate climatic and edaphic variation in hydrological conditions, and fewer species tolerate broader variations in hydrological conditions (Figure 2). Our results also show a higher number of non‐wetland species with narrower climatic hydrological niches (*TP*, *PS*, and *AET*), in comparison with wetland‐adapted species, which had their peak density at wider niche breadths (Figure 3). Consequently, the geographic range size of most Amazonian tree species is relatively narrow (median range size of only 154,000 km^2^) and does not cover more than c.a. 2% of the extent of the Amazon basin (Figure 3). Still, at least 25% of Amazonian tree species are extremely widespread across Neotropical forests, with range sizes twice as large as the Amazon basin (Figure 2, i.e., species above the third quartile for the logarithmic distribution of range size). Taken together, these findings suggest that wetland‐adapted Amazonian tree species have a higher potential for acclimation to both wetter and relatively dryer places, supporting our initial H1 hypothesis (wetland tree species tend to be generalists) in detriment of H2 (wetland tree species tend to be specialists).

Although our findings give support for the hypothesis of more generalist tree species in wetlands than in upland forests, it is worth considering that our method is not able to determine which species are exclusive to each habitat. The species pool of Amazonian wetland forests was built by considering occurrence records that indicated sampling in a wetland habitat (Luize et al., 2018), and so a species will be considered as a “wetland species” even if they have only a few wetland occurrences in the database. The number of habitats a species can thrive is per see one way to estimate its niche breadth (Slatyer et al., 2013) and the inclusion of species that are not exclusive of a given habitat might have inflated the number of species associated with each habitat in our study. Other ways to find species habitat affinities include, for example, indicator species analysis based on abundance records (ter Steege et al., 2013), but these methods still identify the strength of habitat association, not habitat exclusivity. The path forward to reducing uncertainty about the habitat associations of Amazonian tree species requires increased sampling effort for the flora of the region and studies on anatomy and ecophysiology. A rigorous review of tag information in herbarium records by taxonomists (e.g., Cardoso et al., 2017) can also improve our understanding of habitats within Amazonia where the species can thrive.

The positive niche breadth–range size relationships together with the observation of a left‐skewed distribution of hydrological niche breadth indicates that few Amazonian tree species can survive both extremes of the hydrological gradient. This supports previous studies showing that both water surplus and deficit play a prominent role in the evolution of plant physiology and tree species distribution (Esquivel‐Muelbert et al., 2017; Kreft & Jetz, 2007; Moulatlet et al., 2014; Schietti et al., 2014; Silvertown et al., 2015; ter Steege et al., 2003). Hydrological niche segregation between species is expected to act at a very local scale (Silvertown et al., 2015), and findings showing water supply (i.e., water table depth) as the most important predictor of local‐scale distribution of Amazonian tree species support this expectation (Moulatlet et al., 2014; Schietti et al., 2014). However, hydrological niches are also fundamental to defining plant species distribution at very large scales. For instance, dry season length has been shown to be the strongest climatic predictor for the east–west gradient of tree α‐diversity in Amazonia (ter Steege et al., 2003); and the regional and global distribution of vascular plant diversity is driven by water‐energy balance (Kreft & Jetz, 2007; Tavares et al., 2023), emphasizing the coupled effect of seasonal energy input and water supply on plant establishment and coexistence.

Most Amazonian wetlands are forested floodplains, where soils are waterlogged annually from a few days to half of the year, but where seasonal droughts may also take place (Wittmann et al., 2013). This hyper‐seasonality implies that tree species colonizing wetlands need to survive both flooding and droughts during their lifespan (Parolin et al., 2010), and our results demonstrate that this increased tolerance to the contrasting hydrological conditions found in wetlands is very likely to promote wider geographic distributions. Moreover, wetland habitats may act as corridors for tree species dispersal, as wetlands cover large extents of the Neotropics and particularly South America, creating a network of habitat connections both within and among Neotropical biomes. Tolerance to wetland environmental conditions may thus contribute to the explanation of why most tree species occurring in distinct Neotropical biomes have an Amazonian origin (Antonelli et al., 2018). One implication of the larger range sizes found for Amazonian tree species occurring in wetlands is that those tree species are likely to comprise far‐apart isolated populations, and experience genetic divergence through isolation‐by‐distance. Although we did not test for that hypothesis, isolation‐by‐distance has been demonstrated as an influential process acting on *Inga* diversification (Dexter et al., 2017), which is the most species‐rich genus occurring in Amazonian wetland forests (Luize et al., 2018).

The range size of Amazonian tree species has been estimated before (Feeley & Silman, 2009; Gomes et al., 2018; Hubbell et al., 2008; ter Steege et al., 2015, 2016), but the total extent and methodological approach applied to define species distribution differs among studies, precluding further comparisons. To date, the most comprehensive estimate of range sizes for Amazonian plant species was produced with the aim of estimating species extinction risk (Feeley & Silman, 2009). Subsequently, such estimates were applied to investigate broad‐scale macroecological patterns (Dexter & Chave, 2016). The range size measures provided in our study are in accordance with IUCN standards, allowing a better evaluation of their conservation status by offering the extent of occurrences for Amazonian tree species throughout the entire Neotropics, and providing a fast and easily updatable characterization of tree species distribution. Considering our finding that most Amazonian tree species have small range sizes, their conservation status may be worse than previously evaluated (Feeley & Silman, 2009; ter Steege et al., 2015). Furthermore, the α‐convex hull approach has already been used to estimate global range sizes of the Australian seed flora (Gallagher, 2016) and to estimate species richness of the tribe Bignononieae in South America (Meyer et al., 2018), thus offering a common basis for future comparisons.

Our estimates of range size support that at least 25% of the recognized Amazonian tree flora has geographic ranges that extrapolate the limits of the Amazon basin, highlighting the need for biodiversity studies that go beyond Amazonian boundaries if we are to better understand species distributions, abundances, and niche breadths. There is a synergy between species niche breadth and range sizes (Sexton et al., 2017), where the increase in niche breadth translates to an increase in range size, while the occupation of dissimilar environments during range expansion is also likely to broaden the species niches. Additionally, when compared with tree species that only occur in non‐flooded forests, we conclude that tree species occurring in wetlands can “go further” both by being relatively generalist to hydrological conditions and by achieving a widespread geographic area. If we are to move forward our understanding of the origins and maintenance of Neotropical tree diversity, one important functional aspect to be comprehensively considered and described is the species ability to cope with both dry and waterlogged conditions that dominate much of Neotropical forests and savannas.

## AUTHOR CONTRIBUTIONS

**Bruno Garcia Luize:** Conceptualization (lead); data curation (lead); formal analysis (lead); funding acquisition (lead); investigation (lead); methodology (lead); project administration (lead); visualization (lead); writing – original draft (lead); writing – review and editing (lead). **Clarisse Palma‐Silva:** Funding acquisition (equal); investigation (supporting); project administration (supporting); supervision (equal); writing – review and editing (equal). **Tadeu Siqueira:** Formal analysis (supporting); investigation (supporting); methodology (equal); supervision (equal); validation (equal); writing – review and editing (equal). **Thiago Sanna Freire Silva:** Conceptualization (equal); formal analysis (supporting); funding acquisition (equal); investigation (supporting); methodology (supporting); project administration (equal); supervision (equal); validation (equal); visualization (equal); writing – original draft (supporting); writing – review and editing (equal).

## Supporting information


Table S1.

Appendix S1.

## Data Availability

The datasets used for this analysis are freely available and can be accessed online. Species occurrence records are available from the Global Biodiversity Information Facility—GBIF (https://www.gbif.org/occurrence/search) and SpeciesLink (http://inct.splink.org.br/) databases. Climate data are available from WorldClim (https://www.worldclim.org/bioclim). The global dataset of actual evapotranspiration is available from the CGIAR‐CSI GeoPortal (https://cgiarcsi.community/data/global‐high‐resolution‐soil‐water‐balance/). The water table depth model is available upon request from the Authors (Fan et al., 2013). R scripts to compute and export the species range size area and the associated shapefile and to extract and compute the species niche breadth were available.
