# Supplementary material for: Tree species occurring in Amazonian wetland forests consistently show broader range sizes and niche breadths than trees in upland forests
Source: Ecol Evol. 2024 Apr 25;14(4):e11230. doi: 10.1002/ece3.11230 (PMC11045914; doi:10.1002/ece3.11230)
Supplement: Supplementary file 2 — Appendix S1. [file ECE3-14-e11230-s002.docx]

**Appendix**


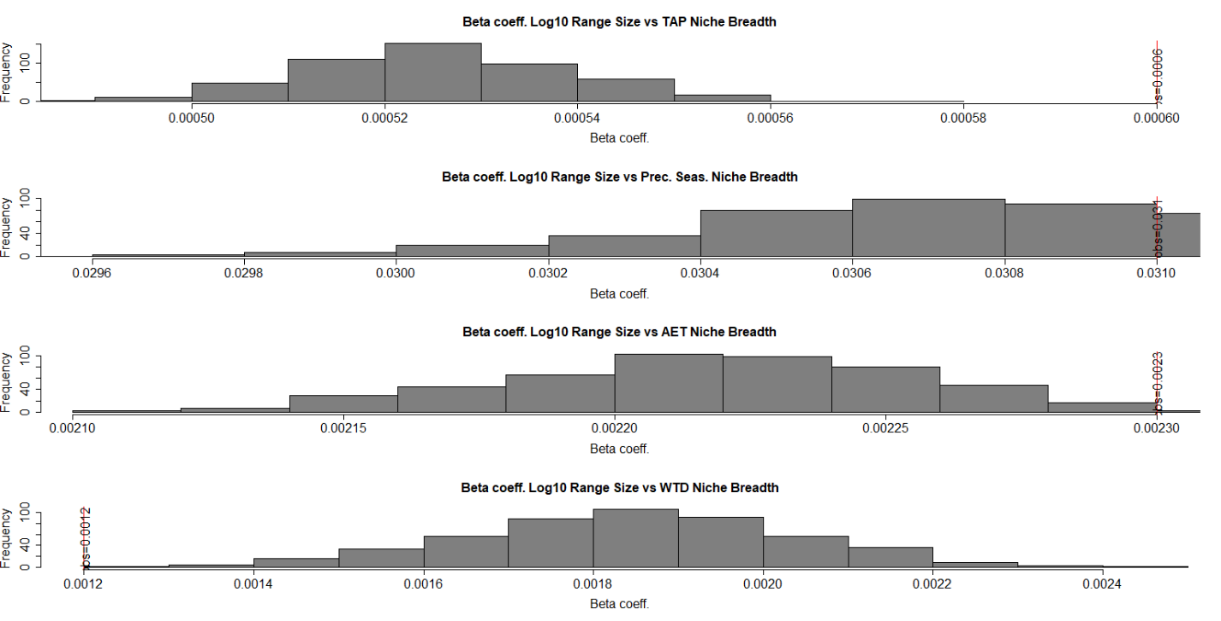


**Figure S1.** Histogram for the slopes estimated by the models ${log}_{10}\left( RS \right)=\alpha+\beta_{1}NB\pm\varepsilon\left( Eq.1 \right)$ relating independent measurements of species niche breadth and range size in comparison with the observed slopes for model relating measurements of species niche breadth and range size computed with all available occurrence records (red line).


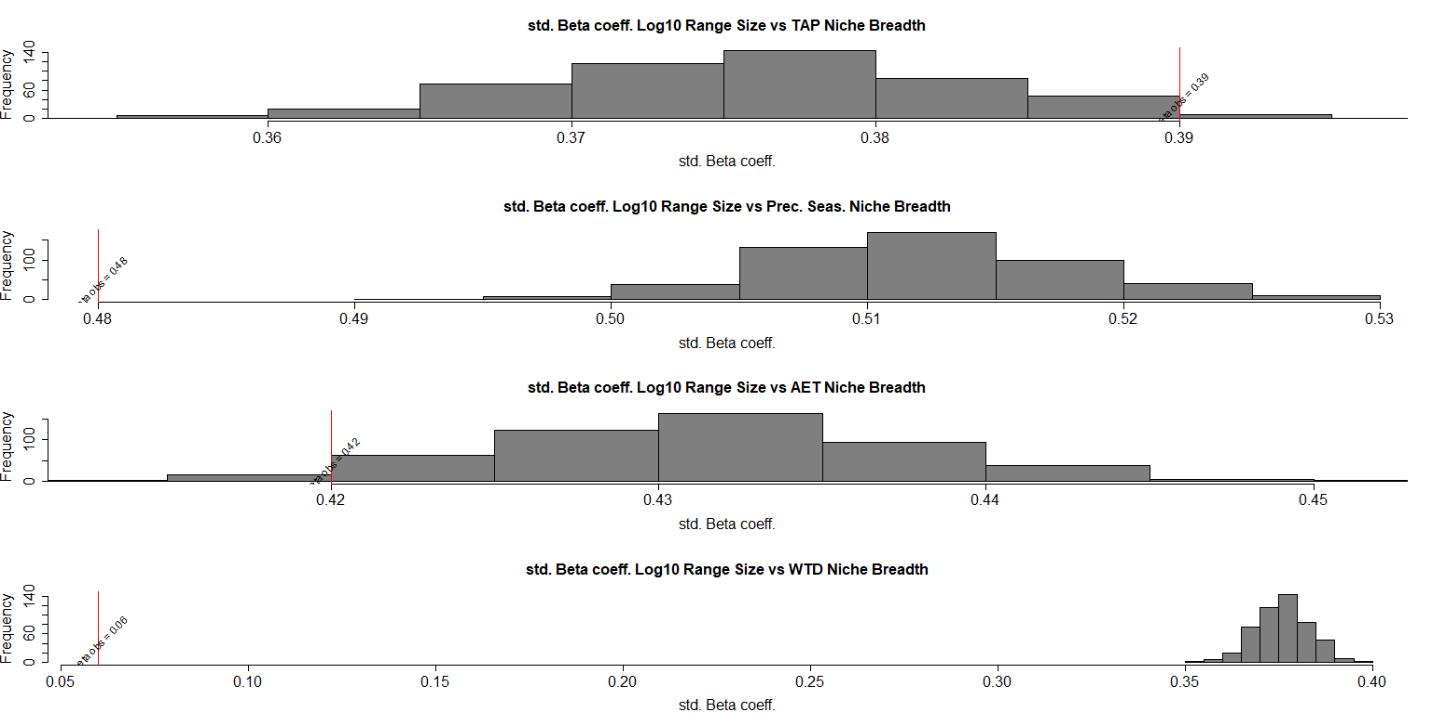


**Figure S2.** Histogram for the standardized slopes estimated by the models ${log}_{10}\left( RS \right)=\alpha+\beta_{1}NB\pm\varepsilon\left( Eq.1 \right)$ relating independent measurements of species niche breadth and range size in comparison with the observed standardized slopes for model relating measurements of species niche breadth and range size computed with all available occurrence records (red line).


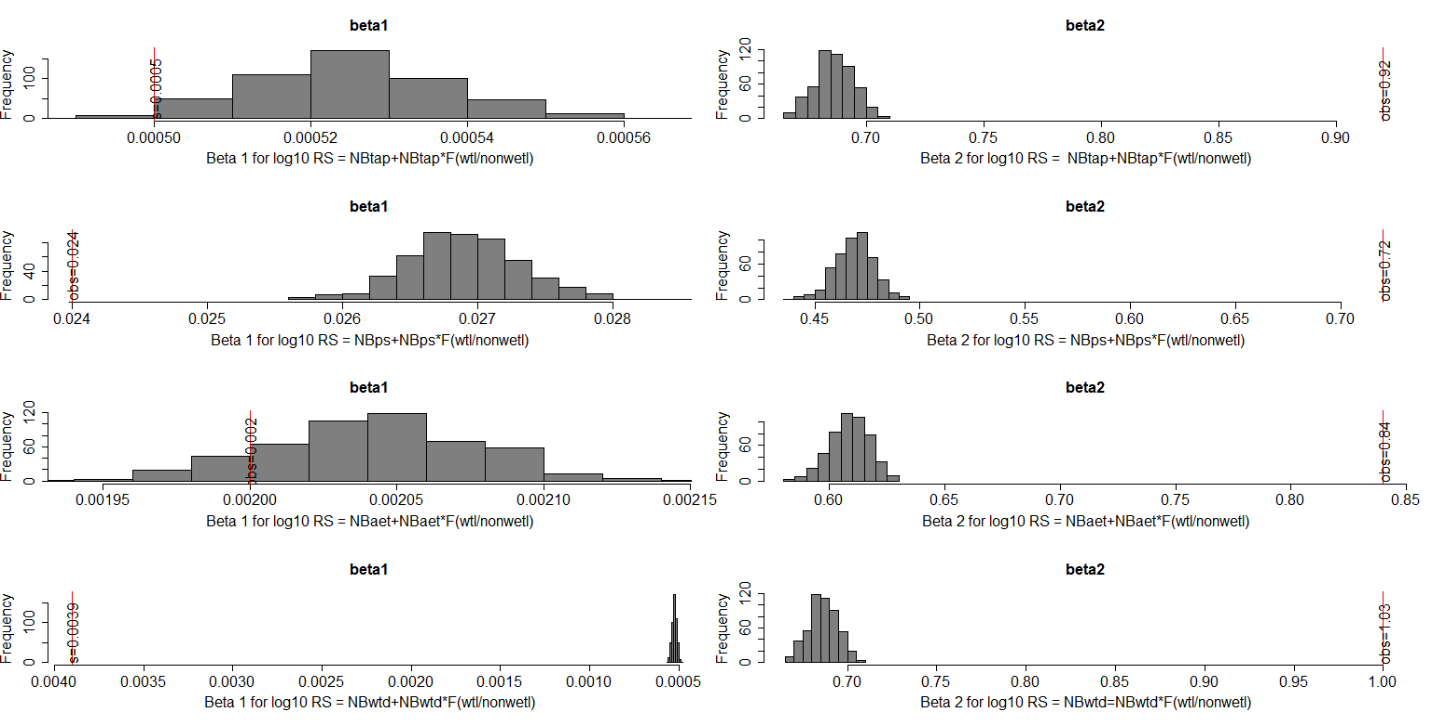


**Figure S3.** Histogram for the slopes estimated by the models ${log}_{10}\left( RS \right)=\alpha+\beta_{1}NB+\beta_{2}\left( W\vee NW \right)\pm\varepsilon\left( Eq.2 \right)$ relating independent measurements of species niche breadth and range size in comparison with the observed slopes for model relating measurements of species niche breadth and range size computed with all available occurrence records (red line).


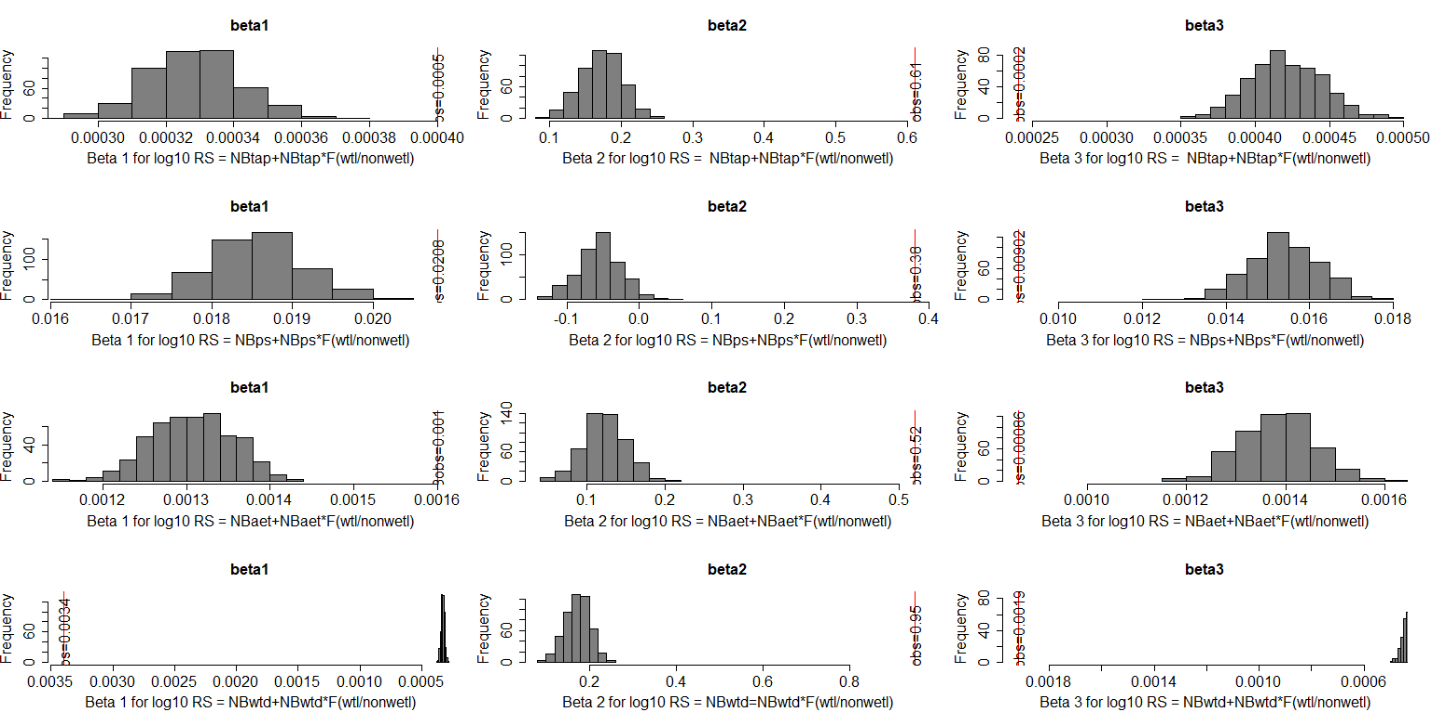


**Figure S4.** Histogram for the slopes estimated by the models (${log}_{10}\left( RS \right)=\alpha+\beta_{1}NB+\beta_{2}\left( NW \right)+\beta_{3}NB\left( W\vee NW \right)\pm\varepsilon\left( Eq.3 \right)$ relating independent measurements of species niche breadth and range size in comparison with the observed slopes for model relating measurements of species niche breadth and range size computed with all available occurrence records (red line).
